# Evaluation of periodontal indices among non-smokers, tobacco, and e-cigarette smokers: a systematic review and network meta-analysis

**DOI:** 10.1007/s00784-022-04531-9

**Published:** 2022-05-13

**Authors:** Paolo Pesce, Maria Menini, Giovanni Ugo, Francesco Bagnasco, Mario Dioguardi, Giuseppe Troiano

**Affiliations:** 1 Department of Surgical Sciences (DISC), University of Genoa, Genoa, Italy; 2 Division of Prosthodontics, Department of Surgical Sciences (DISC), University of Genoa, Genoa, Italy; 3 Department of Clinical and Experimental Medicine, University of Foggia, Foggia, Italy

**Keywords:** Systematic review, Network meta-analysis, Smokers, Tobacco, e-cigarette

## Abstract

**Objectives:**

The detrimental effect of tobacco smoking on periodontal health is well known, while the effect of electronic cigarette on periodontal parameters has been less investigated. The aim of the present systematic review was to compare periodontal indices in three categories of patients: traditional cigarette smokers (TS), e-cigarette smokers (ES), and non-smokers (NS).

**Materials and methods:**

An electronic search was conducted for studies published until December 2021 on MEDLINE (PubMed), ISI Web of Science, and Scopus. A hand search was additionally conducted. Clinical observational and cross-sectional trials investigating periodontal indices among tobacco smokers (TS), electronic cigarette smokers (ES) and non-smokers (NS) were included and selected by 2 independent reviewers. Data on probing depth (PD), plaque index (PI), and bleeding on probing (BOP) were collected. The risk of bias was evaluated according to the NIH quality assessment tool and a network meta-analysis (NMA) was undertaken.

**Results:**

Five relevant studies, from 707 identified, were included. Overall, 512 patients were included, of them 170 were NS, 176 were TS, and 166 were ES. A significant difference in the comparison among TS vs NS: effect size (ES) = 3.297 (95%CI: [2.142–4.454], *p* = 0.001) and TS and ES ES = 2.507 (95%CI: [1.351–3.663], *p* = 0.001) was identified for PD. A significant difference in the comparison among TS and NS, ES = 21.34 (95%CI: [13.41–29.27], *p* = 0.001) and between TS and ES ES = 15.67 (95%CI: [7.73–23.62], *p* = 0.001) was identified for PI. The analysis of BOP values shows a significant difference in the comparison among ES and NS: ES = − 16.22 (95%CI: [− 22.85 to − 9.59], *p* < 0.001) and between TS and NS: ES = − 14.47 (95%CI: [− 21.103 to − 7.848], *p* < 0.001). Based on the SUCRA ranking, NS showed the most favorable outcome for PD and PI, followed by ES. Tobacco smokers were clearly in the last position. Dealing with BoP ES showed the most favorable outcome, followed by TS. NS were in the last position.

**Conclusions:**

Periodontal parameters were similar among NS and ES, while TS presented the worst indices. BoP was reduced both in ES and in TS.

**Clinical relevance:**

Results of the present review suggest a reduced effect on periodontal tissue of e-cig smoking compared to traditional cigarettes, despite recent studies proved that e-cig smoking increases oxidative stress, inflammatory responses, change in pulmonary cellular behavior, and stimulates DNA injury.

## Introduction

Tobacco smoking is known to be associated with serious pathologies including cancer, cardiovascular diseases, chronic bronchitis, emphysema, and several others [1, 2]. Some of them also involves the oral cavity, such as increased risk of developing periodontal disease [3, 4] and more frequent implant failure [5, 6]. Recent investigations confirmed a strong association with worse periodontal status [7, 8] with a greater quantity of plaque and calculus among smokers [2]. This is particularly important considering that more and more clinical studies underscore the paramount importance of oral health to systemic health [9]. Clinical studies have demonstrated both clinical, biochemical, and microbiologic aspects correlating tobacco with the extent and severity of periodontal diseases [3]. In particular, tobacco smoke impairs the protective host response to the bacterial biofilm and, at the same time, increases the production of inflammatory cytokines and enzymes.

Smoke-related lesions can be caused by toxic and irritating substances, but also by high intra-oral temperature, change in pH, and altered immune response [2, 10, 11]. Smoking habit also negatively affects the subgingival microbiome, favoring a more pathogenic profile, depleting the commensal species, and supporting a pathogen-rich community [12, 13].

Additionally, prolonged exposure to cigarette smoke has been reported to impair the growth of human gingival fibroblasts. Smoking increases the formation and accumulation of advanced glycation end products (AGEs) in periodontal tissues [14].

Quitting smoking is usually challenging because nicotine is addictive and can cause withdrawal symptoms such as anxiety and panic disorders [15]. To overcome these problems, over the last decades, a new alternative to tobacco smoking has been introduced: the electronic cigarette, a portable battery-operated device which contains liquid in the tank (usually composed of three main components: a carrier solution (propylene glycol or vegetable glycerin), nicotine (unless a nicotine-free version), and flavoring; which are heated by a resistor releasing an aerosol and inhaled by the user during consumption [2]. In this device, combustion that reaches temperatures of about 800° does not take place; instead, the vaporization of the liquid takes place, at much lower temperatures (about 300 °C), creating less oxidative stress than in traditional cigarettes [2]. It could be assumed that, if existing smokers switched completely from conventional cigarettes (without other changes in usage patterns) to e-cigarettes, there would be a lower burden of disease caused by nicotine addiction [16]. However, the latter are not free from damage: in fact, e-cig have been reported to alter the oral microbiota (in favor of pathogenic germs) exposing to the risk of infection and inflammation and favoring the onset of caries and periodontitis [17]. Additionally, potential risks for cardiovascular and lung health are reported [2].

The aim of the present systematic review was therefore to compare periodontal indices such as PD, PI, and BOP in three categories of patients: TS, ES, and NS.

## Materials and methods

The manuscript was prepared following the Preferred Reporting Items for Systematic Reviews and Meta-Analysis (PRISMA) incorporating network meta-analysis for health care interventions [18] and the protocol was registered with PROSPERO (CRD42021285397). The focused question analyzed in this systematic review was “Is there a difference in periodontal parameters (bleeding on probing (BoP), plaque index (PI), probing depth (PD)) among tobacco smoking, electronic cigarette smoking and non-smoking healthy patients?” The Population, Exposition, Comparison, Outcome (PECO) scheme was taken into consideration in applying eligibility criteria for the focused question.

Population: Healthy patients with no history of periodontists;

Exposition: Use of electronic cigarettes or tobacco smoking;

Comparison: Non-smoking patients;

Outcomes: Periodontal parameters including PD, PI, and BoP.

### Eligibility criteria

Clinical observational and cross-sectional trials investigating periodontal indices among TS, ES, and NS were included. Studies were included only if presented in at least 25 patients in each group and if BoP, PI, and PD values were reported. Traditional cigarette smokers must have been smoking at least 5 cigarettes per day for 1 year. e-cig smokers must have been smoking e-cig exclusively for at least 1 year. Studies with double smokers were excluded. Healthy patients were defined as individuals without systemic diseases (acquired immunodeficiency syndrome, cardiovascular disorders, diabetes, hepatic, and renal disorders). Studies were excluded if patients reported having used antibiotics, non-steroidal anti-inflammatory drugs, and/or steroids within the last 3 months.

Case reports, case series, systematic reviews, animal studies, and in vitro studies and redundant studies were excluded.

### Search strategy

A literature search was carried out using electronic databases (MEDLINE (PubMed), ISI Web of Science, and Scopus). A PubMed search was created and adapted to each database: ((electronic cigarettes) OR (vaping) OR (electronic nicotine delivery systems) OR (e-cigarettes)) AND (dental OR gingiva), and the last electronic search was carried out in December 2021. The references of all the included studies and relevant systematic reviews were screened for additional studies and neither language nor date of publication restriction was adopted.

Additionally, a hand search, based on the number available online since 2000, was conducted in the following journals: *Journal of Periodontology*, *Journal of Clinical Periodontology*, *Clinical Oral Investigations*, *International Journal of Periodontics*, and *Restorative Dentistry*.

### Study selection

Two authors (P. P., G. U.) examined the titles, abstracts, and full texts of the identified articles to check the respect for the inclusion criteria. Cohen’s Kappa was used to assess the inter-examiners agreement. Disagreements were resolved by discussion or by consulting a third reviewer (GT). Full texts of all the eligible articles were downloaded, and in case of exclusion, the reasons for exclusion were registered.

### Data collection

An Excel data sheet (Microsoft Corp.) was used to extract data. Two authors (G. U., P. P.) extracted the following data: study design, country, sponsor, number of smokers and non-smokers, duration of the smoking habit, sex, age, PD, PI, and BOP. If some information was missing, the authors were contacted to obtain that information.

### Risk of bias assessment

Two co-authors (U. G., P. P.) independently assessed the articles according to the NIH quality assessment tool for observational cohort and cross-sectional studies [19] (https://www.nhlbi.nih.gov/health-topics/study-quality-assessment-tools).

Disagreements were resolved by discussing with another co-author (G. T.). The tool is designed to aid the appraisal of the internal validity of cross-sectional, cohort studies, and case-control studies. It comprises 14 criteria and for each domain, the possible answer is yes, no, or other (CD, cannot determine; NA, not applicable; NR, not reported). All responses other than “yes” indicate a risk of bias. Inherent to the design, cross-sectional studies automatically score “not applicable” on criteria 6, 7, 10, and 13.

### Data analysis

After the data extraction process ended, results were pooled together to fit STATA software (network setup command). Relevant assumptions for network meta-analysis were checked, including similarity, transitivity, and consistency [20]. The similarity of included studies was qualitatively assessed, evaluating population, intervention, comparison, and outcome [21]. Transitivity was further assessed by statistically investigating the consistency among the outcomes of direct and indirect comparisons [22]. Hence, network geometry plots and predictive interval plots were created. The network geometry plot was used to illustrate the network of the different groups and analyze connections among them; in this case, we did not disconnect studies nor separate loops. The nodes represent the three groups and edges represent the available direct comparisons between pairs of groups. To estimate the relative ranking of groups using probabilities, surface under the cumulative ranking curves (SUCRA) was used. SUCRA is a simple transformation of the mean rank and is used to provide a hierarchy of the interventions, accounting both for the location and the variance of all relative treatment effects. The larger the SUCRA value, the better the rank of the treatment. A two-tailed *P*-value of 0.05 was considered significant for hypothesis testing. Information regarding the mean difference, SD, category of subjects, and the number of subjects was extracted from clinical studies. Three separate network meta-analyses were undertaken for PI, PD, and BOP.

## Results

### Study selection

**Fig. 1:**
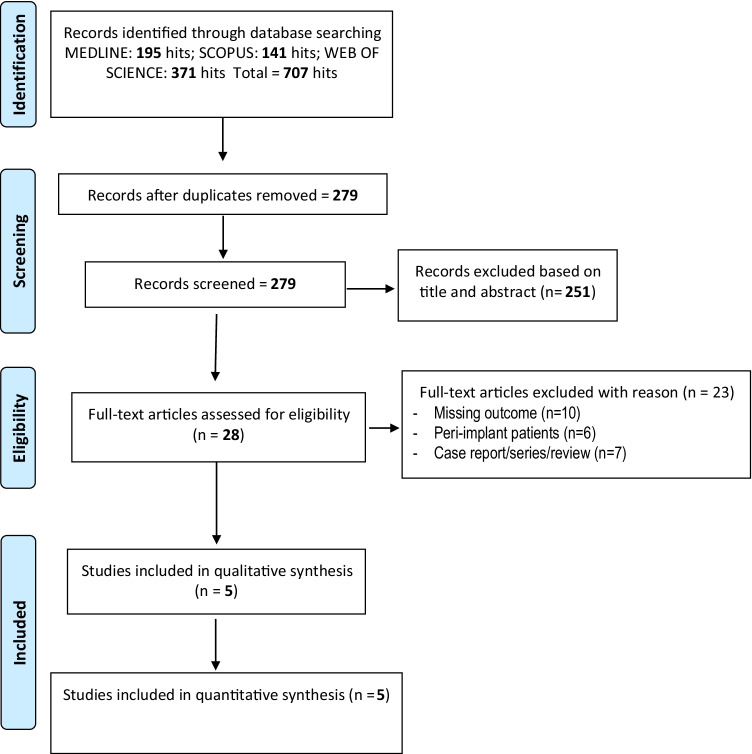
Flowchart of the included studies

The flowchart of the selection process is reported in Fig. 1. Seven hundred seven studies were identified by the search strategy (195 in Medline, 141 Scopus, and 371 Web of Science) and after the duplicate removal 279 were screened. After titles and abstracts evaluation, 251 studies were eliminated, and 28 full texts were downloaded.

After full-text evaluation, 23 studies were excluded, and the reasons for exclusion are reported in Fig. 1. The kappa value for the inter-reviewer agreement was 0.93 indicating very good agreement. Five articles were included [14, 23–26], and the main characteristics are described in Tables 1, 2, and 3. Three authors were contacted in order to ask for additional information and all of them answered [23–25].

**Table 1:** Main information on the included studies - NS

Pub year	Title	Country	NF total	M/F	Age	Standard deviation	PI non-smokers %	Standard deviation	BOP non-smokers %	Standard deviation	PD non-smokers (mm)	Standard deviation
2017	Comparison of periodontal parameters and self-perceived oral symptoms among cigarette smokers, individuals vaping electronic cigarettes, and never-smokers	Saudi Arabia	30	Male	40,7	1,6	21,4	2.8	27.5	3.2	0.57	0.03
2018	Clinical and radiographic periodontal status and whole salivary cotinine. IL-1β and IL-6 levels in cigarette- and waterpipe-smokers and E-cig users	Saudi Arabia	38	Male	41.6	4.5	23	0.004	37.4	6.4	1.4	0.1
2018	Impact of cigarette smoking and vaping on the outcome of full-mouth ultrasonic scaling among patients with gingival inflammation: a prospective study	Saudi Arabia	31	Male	32.6	3.5	46.3	5.2	38.2	6.5	4.2	0.3
2019	Clinical periodontal status and gingival crevicular fluid cytokine profile among cigarette-smokers. electronic-cigarette users and never-smokers	Saudi Arabia	45	124 males/11females	40.6	3.3	18.2	2.45	28.4	1.61	1.6	0.23
2020	Comparison of self-rated oral symptoms and periodontal status among cigarette smokers and individuals using electronic nicotine delivery systems	Saudi Arabia	26	Male	33.5	1.4	4.2	0.5	22.1	3.3	1.5	0.2

**Table 2:** Main information on the included studies - TS

Pub year	Title	FUM total	Age	Standard deviation	Duration of habit (years)	Standard deviation	Daily frequency	Standard deviation	PI smokers %	Standard Deviation	BOP smokers %	Standard Deviation	PD smokers (mm)	Standard Deviation
2017	Comparison of periodontal parameters and self-perceived Oral symptoms among cigarette smokers. individuals vaping electronic cigarettes. and never-Smokers	33	41.3	2.8	5.4	1.6	13.3	2.6	52.1	6.6	5.8	0.8	6.52	1.3
2018	Clinical and radiographic periodontal status and whole salivary cotinine. IL-1β and IL-6 levels in cigarette- and waterpipe-smokers and E-cig users	39	42.4	5.6	17.2	2.5	16.2	2.5	49.1	0.4	19.5	4.2	4.6	0.5
2018	Impact of cigarette smoking and vaping on the outcome of full-mouth ultrasonic scaling among patients with gingival inflammation: a prospective study	30	36.4	2.8	10.4	1.8	9.3	4.6	49.4	7.3	17.2	3.3	5.2	0.4
2019	Clinical periodontal status and gingival crevicular fluid cytokine profile among cigarette-smokers. electronic-cigarette users and never-smokers	46	44.2	± 3.5	14.2	± 0.6			42.1	1.36	10.6	1.56	5.3	0.43
2020	Comparison of self-rated oral symptoms and periodontal status among cigarette smokers and individuals using electronic nicotine delivery systems	28	33.3	1.2	6.1	0.5			39.3	8.2	12.1	3.6	4.2	0.2

**Table 3:** Main information on the included studies - ES

Pub year	Title	Study design	Country	ELEC total	Age	Standard deviation	Duration of habits (years)	Standard deviation)	Daily frequency	Standard deviation	PI e-cig smokers %	Standard Deviation	BOP e-cig smokers %	Standard deviation	PD e-cig smokers (mm)	Standard deviation
2017	Comparison of periodontal parameters and self-perceived oral symptoms among cigarette smokers. Individuals vaping electronic cigarettes and never-smokers	Comparative study	Saudi Arabia	31	37.6	2.1	2.2	0.2	6.8	0.8	23.3	3.4	4.6	2.9	2.82	0.07
2018	Clinical and radiographic periodontal status and whole salivary cotinine. IL-1β and IL-6 levels in cigarette- and waterpipe-smokers and E-cig users	Comparative Study	Saudi Arabia	37	29.3	1.6	3.1	0.4	9.2	1.4	43.5	0.08	16.4	2.5	1.8	0.2
2018	Impact of cigarette smoking and vaping on the outcome of full-mouth ultrasonic scaling among patients with gingival inflammation: a prospective study	Prospective study	Saudi Arabia	28	32.5	4.8	3.1	0.4	12.5	0.8	43.5	5.6	11.6	4.5	4.6	0.2
2019	Clinical periodontal status and gingival crevicular fluid cytokine profile among cigarette-smokers. electronic-cigarette users and never-smokers	Comparative Study	Saudi Arabia	44	36.5	1.7	9.4	2.6			33.4	2.3	12.2	1.39	2.5	0.27
2020	Comparison of self-rated oral symptoms and periodontal status among cigarette smokers and individuals using electronic nicotine delivery systems	Comparative Study	Saudi Arabia	26	31.6	2.4	0.9	0.2	30.2	8.5	25.6	6.2	11.5	0.8	1.5	0.3

Overall, 512 patients were included, of them 170 were non-smokers, 176 were traditional cigarette smokers, and 166 were e-cig smokers. Almost all the included patients were men (501). The mean age of the non-smokers was 37.8, the mean age of tobacco smokers was 39.52, and the mean age of e-cig smokers was 33.5. The mean duration of the habit was 10.66 years for the tobacco smokers and 3.74 years for the e-cig smokers.

### Risk of bias

The risk of bias among the included studies is reported in Table 4. In our evaluation, all the studies included were of good or fair methodological quality. Specific limitations included the lack of information on the population included and no evidence of adjustment for confounders.

**Table 4:** Risk of BIAS evaluation

	Fawad et al.	Sameer et al.	Shatha et al.	Munerah et al.	Fahim et al.
1. Was the research question or objective in this paper clearly stated?	Y	Y	Y	Y	Y
2. Was the study population clearly specified and defined?	Y	N	Y	Y	Y
3. Was the participation rate of eligible persons at least 50%?	Y	CD	CD	CD	CD
4. Were all the subjects selected or recruited from the same or similar populations (including the same time period)? Were inclusion and exclusion criteria for being in the study prespecified and applied uniformly to all participants?	Y	CD	CD	CD	CD
5. Was a sample size justification, power description, or variance and effect estimates provided?	Y	Y	Y	Y	Y
6. For the analyses in this paper, were the exposure(s) of interest measured prior to the outcome(s) being measured?	NA	NA	Y	NA	NA
7. Was the timeframe sufficient so that one could reasonably expect to see an association between exposure and outcome if it existed?	NA	NA	Y	NA	NA
8. For exposures that can vary in amount or level, did the study examine different levels of the exposure as related to the outcome (e.g., categories of exposure, or exposure measured as continuous variable)?	NA	NA	NA	NA	NA
9. Were the exposure measures (independent variables) clearly defined, valid, reliable, and implemented consistently across all study participants?	Y	Y	Y	Y	Y
10. Was the exposure(s) assessed more than once over time?	NA	NA	Y	NA	NA
11. Were the outcome measures (dependent variables) clearly defined, valid, reliable, and implemented consistently across all study participants?	Y	Y	Y	Y	Y
12. Were the outcome assessors blinded to the exposure status of participants?	Y	Y	Y	N	Y
13. Was loss to follow-up after baseline 20% or less?	NA	NA	Y	NA	NA
14. Were key potential confounding variables measured and adjusted statistically for their impact on the relationship between exposure(s) and outcome(s)?	N	N	N	N	N

*CD*, cannot determine; *NA*, not applicable; *NR*, not reported

### Synthesis of the results

**Fig. 2:**
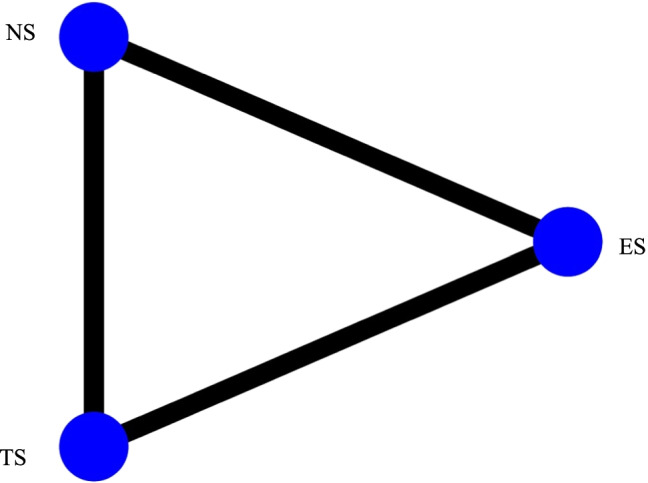
Network geometry plot

Figure 2 illustrates the network geometry plot for the analyzed outcomes. The size of the blue circles is proportional to the sample size for any group, while the thickness of the lines connecting the two circles is proportional to the number of studies comparing the groups. The geometry plot is the same for all three analyzed outcomes. In all the comparisons, the absence of inconsistency was assessed by both global and local tests. The evidence reported was all obtained by direct comparisons since all the studies showed three groups.

#### Probing depth

**Fig. 3:**
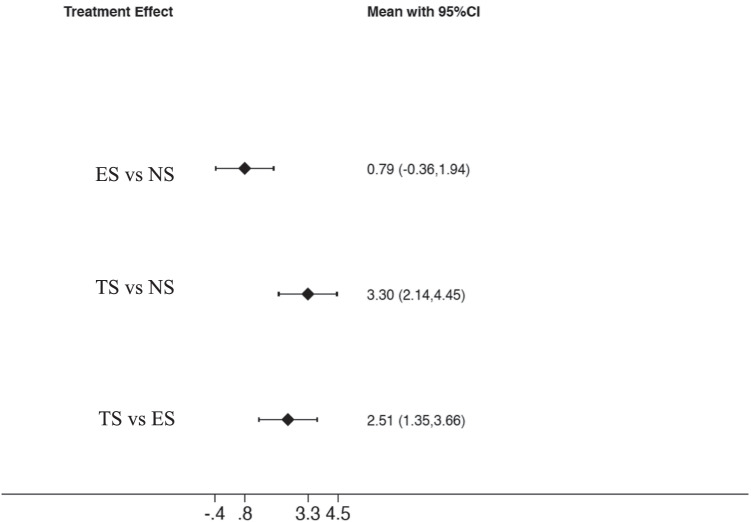
Interval plot for probing depth

**Fig. 4:**
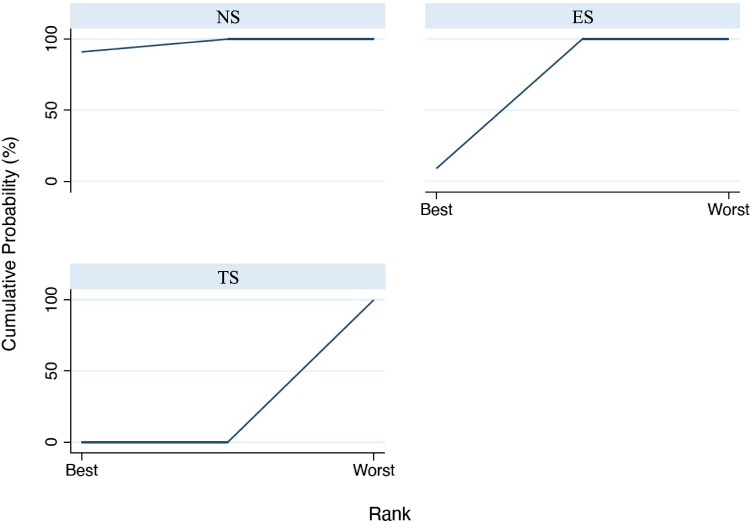
SUCRA ranking for probing depth

The first analyzed outcome was PD. Figure 3 reports the interval plot for the analyzed outcome; in particular, the width of confidence intervals (black horizontal lines) indicates the amount of variance for the specific comparison and seems to be constant for all three comparisons. Results of the consistency model revealed a significant difference in the comparison among TS vs NS: effect size (ES) = 3.297 (95%CI: [2.142 to 4.454], *p* = 0.001), while for the comparison between TS and ES was ES = 2.507 (95%CI: [1.351 to 3.663], *p* = 0.001), while a non-significant difference was present between ES and NS ES = 0.791 (95%CI: [− 0.360 to 1.942], *p* = 0.178). Based on the SUCRA ranking (Fig. 4), NS showed the most favorable outcome for PD, followed by ES. Tobacco smokers were clearly in the last position.

#### Plaque index

**Fig. 5:**
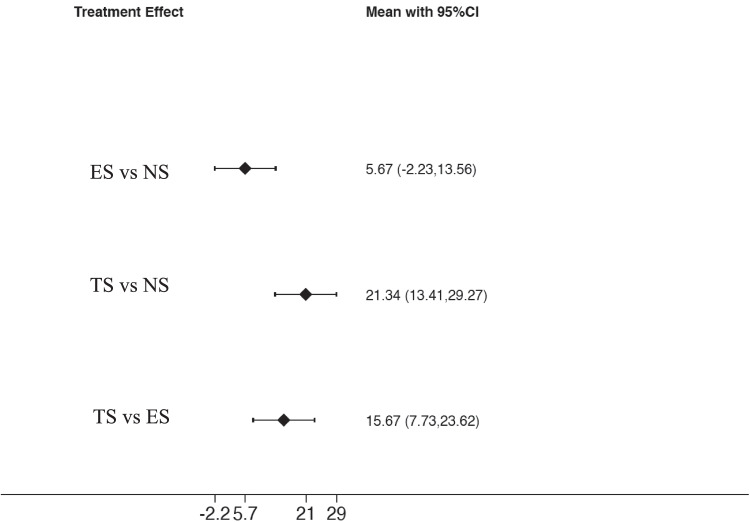
Interval plot for plaque index

**Fig. 6:**
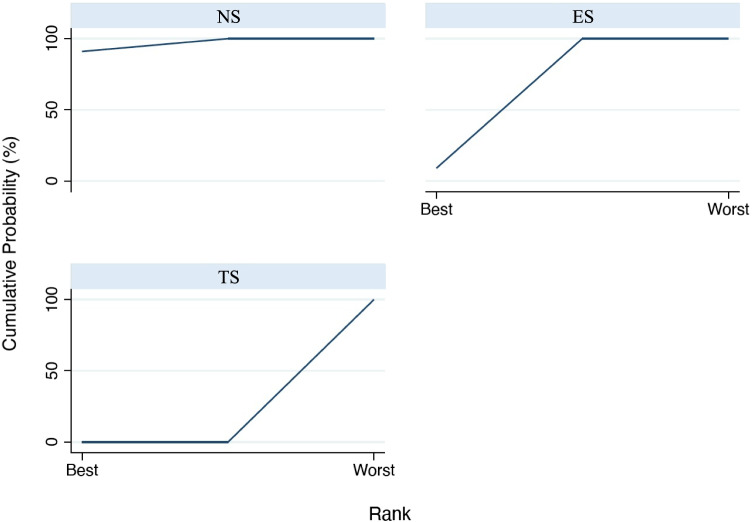
SUCRA ranking for plaque index

The analysis of PI values (Fig. 5) demonstrates a significant difference in the comparison among TS and NS: ES = 21.34 (95%CI: [13.41 to 29.27], *p* = 0.001), while a non-significant difference was present among ES and NS: ES = 5.67 (95%CI: [− 2.23 to 13.56], *p* = 0.160). In addition, the comparison between TS and ES was also significant ES = 15.67 (95%CI: [7.73 to 23.62], *p* = 0.001). Based on the SUCRA ranking (Fig. 6), NS showed the most favorable outcome for PI, followed by ES. Tobacco smokers were clearly in the last position.

#### Bleeding on probing

**Fig. 7:**
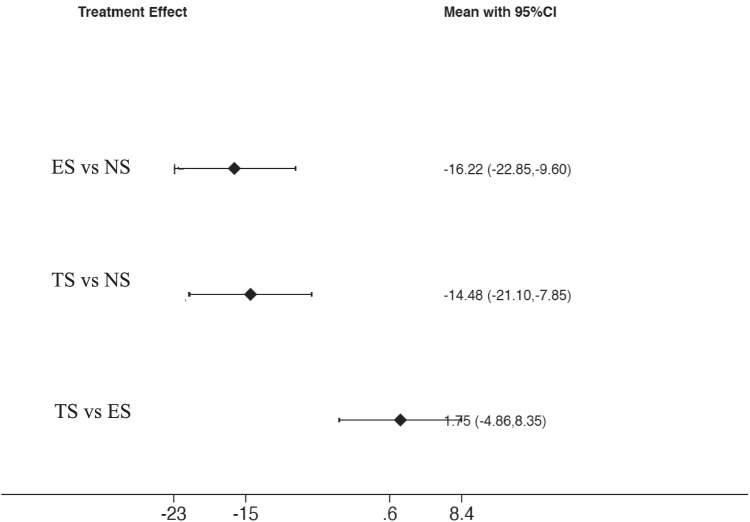
Interval plot for bleeding on probing

**Fig. 8:**
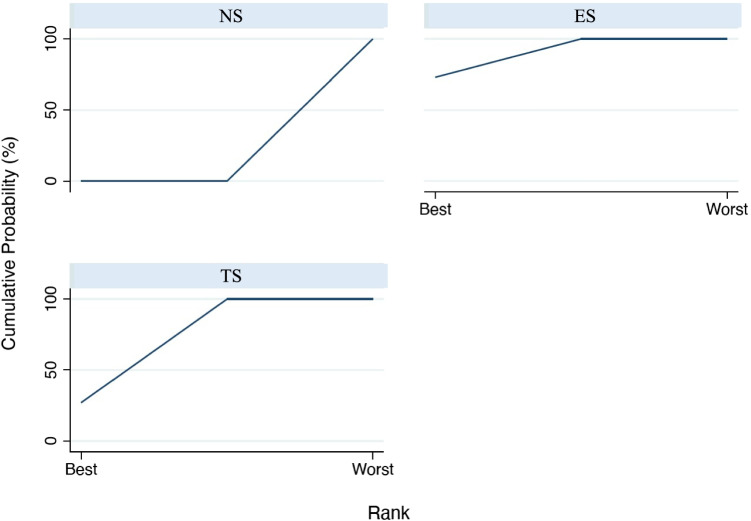
SUCRA ranking for bleeding on probing

The analysis of BOP values (Fig. 7) among the included studies demonstrates a significant difference in the comparison among ES and NS: ES = − 16.22 (95%CI: [− 22.85 to − 9.59], *p* < 0.001) and between TS and NS: ES = − 14.47 (95%CI: [− 21.103. to − 7.848], *p* < 0.001). Conversely, a non-significant difference was present between TS and ES: ES = 1.747 (95% CI [− 4.859 to 8.353], *p* = 0.604). Based on the SUCRA ranking (Fig. 8), ES showed the most favorable outcome for BOP, followed by TS. NS were in the last position.

## Discussion

The results of the present systematic review suggest that traditional cigarette smokers (TS) present worse conditions than the other groups (non-smokers (NS) and electronic cigarette smokers (ES)). TS patients presented a worse plaque index, less bleeding on probing (despite a higher plaque index), which is second to e-cigarette smokers, and mean probing depth was often above the physiological level (3 mm [27]). This confirms that the habitual use of tobacco products increases oxidative stress in periodontal tissues and, if left untreated or uncontrolled, can contribute to periodontal disease and alveolar bone loss [24, 28].

On the other hand, e-cig smokers presented a worst periodontal situation than non-smokers, with higher PI and PD, but with lower values when compared to traditional cigarette smokers. The higher percentage of PI among tobacco smokers may correspond to the neglected attitude toward oral hygiene care. However, it is interesting to underline that ES showed a lower BOP than TS, despite having a greater PI than TS. NS had the worst BOP values. The suppressive effect of tobacco smoking on gingival bleeding is well known [29], even if the mechanisms by which smoking reduces bleeding on probing are not completely understood. It is proved that nicotine causes acute vasoconstriction in human skin [30] and that the association between smoking and gingival bleeding is dose-dependent with a plateau effect approximately 10 to 20 cigarettes per day [29]. On the other hand, the higher suppressive effect on bleeding of e-cig is not yet understood even if this can be attributed to the presence of nicotine in e-cig liquid [31]. Recent studies proved that e-cig smoking increases oxidative stress, inflammatory responses, change in pulmonary cellular behavior, and stimulates DNA injury [32, 33]. Additionally in vitro tests demonstrate that the flavoring agents that are combined with the aerosol of e-cig have been shown to enhance DNA injury and upregulation of several inflammatory proteins such as cyclooxygenase and prostaglandin E2 in gingival cells [34, 35].

It should be acknowledged that several factors may have influenced the current results: firstly, the daily frequency of nicotine inhalation was nearly twice as high among traditional smokers than among e-cigarette smokers. Also, the duration of the vaping habit was relatively shorter. In fact, individuals who smoked e-cig had this habit for less time than traditional smokers. Furthermore, the participants were relatively young. It is known that there is a dose-dependent and dose-duration-dependent relationship between smoking and periodontal disease [36]. Therefore, individuals with a longer history (> 5 years) and a higher daily frequency of vaping (> 15 times a day) are hypothesized to be more susceptible to periodontal inflammation than individuals with a shorter history (< 5 years) and less frequency of e-cig vaping (< 10 times a day) as the sample reported in the present systematic review [24]. Furthermore, three studies included among the exclusion criteria e-cig smokers with a previous history of tobacco smoking [14, 24, 25]; on the contrary, that information was not available in two studies, and this could have influenced the results. Additionally, the majority of the included patients were men since only one study considered women [23] and in two studies the average age was about 10 years lower than the others [14, 26], and this could have affected the clinical outcomes and the level of damage on the periodontium. Observed differences in the outcomes among the three groups may be attributable to differences in demographic composition (sex, age, socioeconomic position) or other prognostic factors even if strict criteria to define TS and ES were used and double smokers and individuals with systemic diseases such as diabetes mellitus were excluded from the present work to reduce possible confounding factors. Furthermore, all included studies were conducted in Saudi Arabia and this fact may limit the generalization of the findings as the traditional and/or racial characteristics such as diet and life conditions are likely to affect the clinical findings. Although evidence from this network meta-analysis comes mainly from direct evidence requiring a lower power to draw conclusions, the number of studies and patients included is still low encouraging the performance of further studies in the future.

## Conclusions

Within the limits of the present study, periodontal parameters were similar among NS and ES, while TS presented the worst indices. BoP was reduced both in ES and in TS compared to NS. Well-designed long-term clinical trials/studies are needed to confirm these results.
